# Symmetry breaking during homodimeric assembly activates an E3 ubiquitin ligase

**DOI:** 10.1038/s41598-017-01880-4

**Published:** 2017-05-11

**Authors:** Zhaofeng Ye, Patrick G. Needham, Samuel K. Estabrooks, Susan K. Whitaker, Brandon L. Garcia, Saurav Misra, Jeffrey L. Brodsky, Carlos J. Camacho

**Affiliations:** 1 Department of Computational and Systems Biology, University of Pittsburgh, Pittsburgh, PA 15206 USA; 2 Department of Biological Sciences, University of Pittsburgh, Pittsburgh, PA 15206 USA; 3 Department of Biochemistry and Molecular Biophysics, Kansas State University, Manhattan, KS 66506 USA; 4 School of Medicine, Tsinghua University, Beijing, 100084 China

## Abstract

C-terminus of Hsc/p70-Interacting Protein (CHIP) is a homodimeric E3 ubiquitin ligase. Each CHIP monomer consists of a tetratricopeptide-repeat (TPR), helix-turn-helix (HH), and U-box domain. In contrast to nearly all homodimeric proteins, CHIP is asymmetric. To uncover the origins of asymmetry, we performed molecular dynamics simulations of dimer assembly. We determined that a CHIP monomer is most stable when the HH domain has an extended helix that supports intra-monomer TPR-U-box interaction, blocking the E2-binding surface of the U-box. We also discovered that monomers first dimerize symmetrically through their HH domains, which then triggers U-box dimerization. This brings the extended helices into close proximity, including a repulsive stretch of positively charged residues. Unable to smoothly unwind, this conflict bends the helices until the helix of one protomer breaks to relieve the repulsion. The abrupt snapping of the helix forces the C-terminal residues of the other protomer to disrupt that protomer’s TPR-U-box tight binding interface, swiftly exposing and activating one of the E2 binding sites. Mutagenesis and biochemical experiments confirm that C-terminal residues are necessary both to maintain CHIP stability and function. This novel mechanism indicates how a ubiquitin ligase maintains an inactive monomeric form that rapidly activates only after asymmetric assembly.

## Introduction

Generally, the lowest energy state of protein assembly is symmetrical, whereas asymmetry is associated with energy frustration and structural instability^1^. However, symmetry is not evolutionarily constrained, and many oligomeric enzymes are known to be asymmetric with only half-of-sites active^2–5^. In the majority of such oligomers, asymmetry results from conformational changes triggered by ligand binding^2, 6, 7^. Another mechanism to fold and activate asymmetric dimers requires that one of the ligand-binding sites is deformed^3, 6, 7^. However, very few of the vast number of known homo-multimeric proteins assemble into asymmetric structures^6^. Among the exceptions are the C-terminus of Hsc/p70-Interacting Protein (CHIP)^8^, an E3 ubiquitin ligase that associates with cyoplasmic Hsp70 and Hsp90 chaperones, and Hikeshi^9^, a nuclear import protein that also binds Hsp70. Unveiling the mechanism of symmetry breaking in homo-oligomers will shed light on new principles of folding and assembly for this important class of proteins.

**Figure 1:**
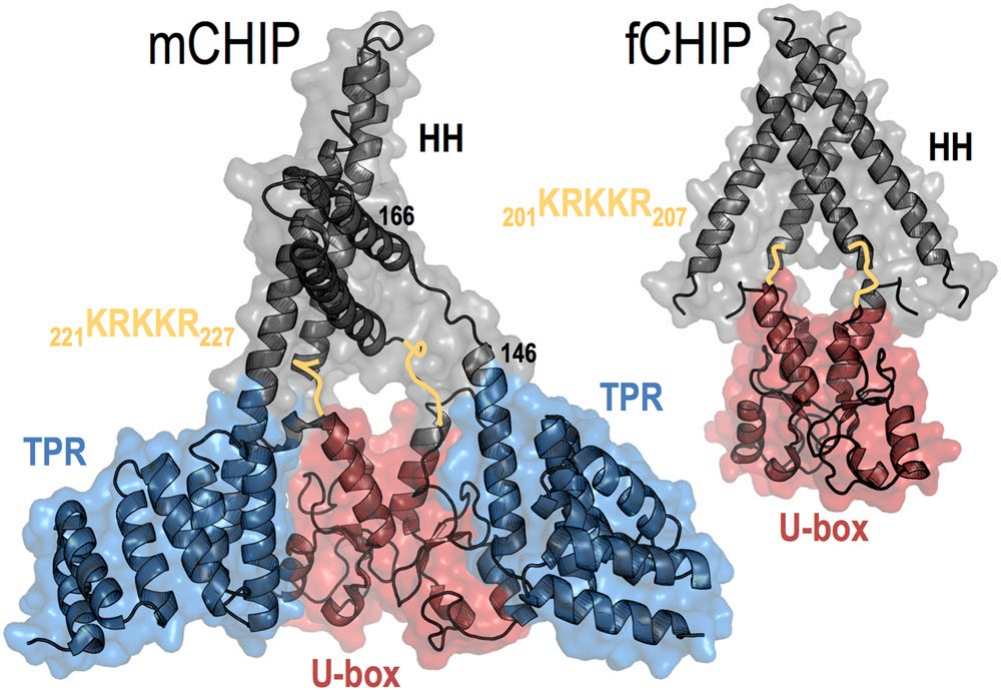
Crystal structures show asymmetric CHIP (mouse; PDB 2C2L) and symmetric fCHIP (Zebrafish; PDB 2F42) without the TPR domain. Blue mesh depicts the TPR domains. Grey mesh indicates the helix-turn-helix (HH) dimers. Red mesh shows U-box dimers. Gold lines highlight the basic residues 221-to-227 linking the HH domains and the U-boxes.

The CHIP homodimer consists of three domains: tetratricopeptide repeat (TPR), helix-turn-helix (HH), and U-box^8^ (Fig. 1). CHIP targets misfolded, chaperone-bound substrates for proteasomal degradation by transferring ubiquitin from a compatible E2 ubiquitin conjugating-enzyme, which associates with the U-box domain, to a lysine residue on the target protein^10, 11^. The crystal structure of murine CHIP (CHIP, PDB: 2C2L), which differs from human CHIP by one residue at the N-terminus, showcases an asymmetric homodimer in which only one U-box domain has an accessible E2-binding surface. The E2 binding site/U-box in the second protomer is blocked by intra-protomer packing against the TPR domain^8^ (Fig. 1). In this second protomer, the HH domain is bent, whereas the HH domain of the first protomer is extended. This pronounced asymmetry contrasts strongly with the symmetric structure of a Zebrafish CHIP construct (fCHIP, PDB: 2F42) from which the TPR domain is deleted^12^ (Fig. 1).

CHIP plays essential roles in modulation of the stress response, protein quality control, innate immune signaling, neurodegenerative disorders, and cancer progression^13–20^. In addition to revealing general assembly principles of asymmetric heterodimerization, a definition of the CHIP folding pathway may uncover the nature of disease-causing mutations in this critical proteostasis component (see e.g. ref. 21) and inform the development of small molecule therapeutics. Here, we describe for the first time the origin of symmetry breaking in homodimerization. We define the CHIP dimer assembly pathway using full atom molecular dynamics simulations (MDS), site-directed mutagenesis, and biochemical studies. Our analyses indicate that the CHIP monomer is most stable when the HH domain has an extended helix that supports domain-domain interaction between the U-box and TPR, which blocks the E2-binding site. Activation requires that this interaction be disrupted in at least one protomer of the CHIP dimer. We reveal how the assembly of the asymmetric dimer leads to kinetic (rather than thermodynamic) disruption of U-box and TPR domain packing in one of the protomers, which exposes the E2 binding site. Specifically, two monomers initially dimerize symmetrically through their HH domains. This initial assembly event triggers U-box dimerization, which unites positively charged residues from the linker regions between the U-box and the HH domains of each protomer. In turn, this repulsion is resolved by breaking a helix in one of the protomers. This action forces the opposite protomer to wedge its C-terminal through its own TPR and U-box domain-domain interface in tens of nanoseconds, forming a stable asymmetric CHIP dimer^8^. We also confirmed that in the absence of the TPR domain, CHIP folds into a symmetric structure, as observed in the crystal structure of CHIP lacking the TPR domain^12^. Simulations, enzyme activity assays, and biophysical methods further validate that a short C-terminal segment plays a critical role in stabilizing the active, asymmetric CHIP dimer. Our findings describe a novel assembly mechanism that transduces the snapping of a helix into a mechanical force that kinetically activates the CHIP dimer by disrupting a stable domain-domain interaction. This model delineates how a ligand- and chaperone-dependent ubiquitin ligase is maintained in an inactive monomeric form that is rapidly activated only after assembly.

## Results

### U-box-TPR interdomain packing is favored in the CHIP monomer

**Figure 2:**
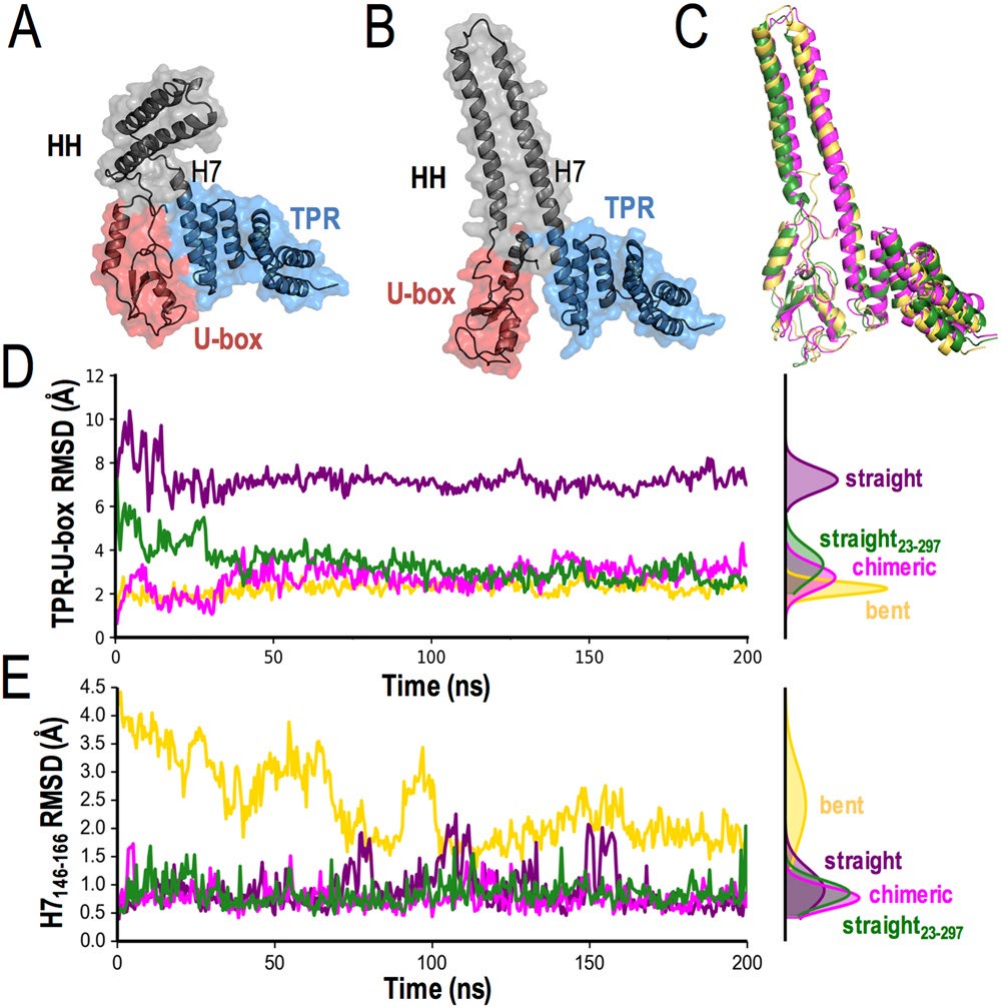
Chimera of extended helix 7 and TPR/U-box complex is more stable than bent or straight CHIP monomers. Structures of (**A**) bent and (**B**) straight monomers from the CHIP crystal structure. The bent monomer has helix 7 (H7) between HH and TPR domains broken, and TPR/U-box bound with the E2 binding site buried. The straight monomer has an extended H7, and the TPR and U-box are separated by the C-terminus to expose an E2 binding site. (**C**) Structural alignments of MDS snapshots of bent monomer (gold) and truncated straight_23–297_ monomer (missing last six C-terminal residues) (forest) to chimeric monomer (magenta) yield an overall backbone RMSD of 2.7 and 3.3 Å. Backbone RMSD relative to the crystal structure of (**D**) the TPR/U-box complex and (**E**) the H7 between residues 146–166 as a function of time. The bent, straight, chimeric and truncated straight_23–297_ RMSD distributions are shown in gold, purple, magenta and forest, respectively.

Crystallographic studies show that full-length murine CHIP (“CHIP”) forms an asymmetric dimer^8^ (Fig. 1). In one of the protomers (referred to as “bent”), the HH domain and TPR-U-box complex are separated by a broken helix 7 (H7). In this protomer configuration, a tight interface formed by the U-box and TPR domains obscures the E2 binding site (Fig. 2A). In the other protomer (referred to as “straight”) H7 is extended and contiguous with the TPR domain, which is separated from the U-box domain by the protomer’s C-terminus. The E2 binding site of the U-box is surface-exposed and accessible in this protomer (Fig. 2B).

To gain insight into the structures of monomeric CHIP, we ran MDS of isolated bent and straight monomers in explicit solvent. Simulations of the bent monomer show that packing between the TPR and U-box domains remains stable (Fig. 2D) and H7 is capable of sampling an extended helix connected to the HH domain, similar to the straight monomer (Fig. 2E). Simulations of the straight monomer show that the extended H7 is maintained (Fig. 2E), and the C-terminus remains inserted between the U-box and TPR domains throughout (Fig. 2D).

To further investigate the stability of the straight monomer, we deleted its six C-terminal residues and performed MDS of the truncation, CHIP_23–297_. In the absence of the C-terminal residues, H7 is stable. However, a domain-domain interaction between the TPR and U-box can form and assumes a conformation similar to that of the bent monomer of the asymmetric CHIP dimer (Fig. 2D,E). The stability of the chimeric structure formed by the truncated straight CHIP_23–297_ (Fig. 2C) was further examined in full length (minus disordered N-terminal) CHIP_23–303_. Interestingly, this chimera combines the stable domains from each monomeric structure, i.e., the extended helix from the straight monomer and the TPR-U-box complex from the bent monomer. Our simulations show that the *chimeric* monomer is more stable than either the straight or bent monomer (Fig. 2D,E), and is readily sampled by both the bent monomer and the truncated straight monomer (Fig. 2C). Folding free energy calculations using MM/PBSA^22^ further support our conclusion that the chimera is the most stable monomeric structure (Supplementary Fig. S1).

### Models of nucleation events during CHIP dimerization

While the structure of the CHIP dimer is asymmetric overall, the HH and U-box dimers within this structure are symmetric (Fig. 1). Computational estimates^23^ suggest that the HH and U-box dimerization interfaces have similar interaction energies (ΔG_int_) of −15.2 and −14.4 kcal/mol, respectively. The HH dimer is relatively rigid and dominated by hydrophobic interactions (the hydropobic-polar residues ratio is ~2:1), whereas the U-box dimer is more flexible and mediated primarily by electrostatic interactions (hydropobic-polar residues ratio is ~1:1). We did not observe interconversions between the bent and straight monomers within the time scale of our simulations. Such interconversions are expected to be quite slow, since large thermal fluctuations are required to disrupt TPR-U-box packing in the bent monomer (interaction energy estimate ΔG_int_ = −20 kcal/mol^23^). This suggests that the dimer asymmetry arises through an induced-fit process initiated by dimer formation. To test this idea, we built two different dimeric assemblies for the bent, straight, and chimeric monomer models, employing either “HH-dimer-first” or “U-box-dimer-first” dimerization modes.

**Figure 3:**
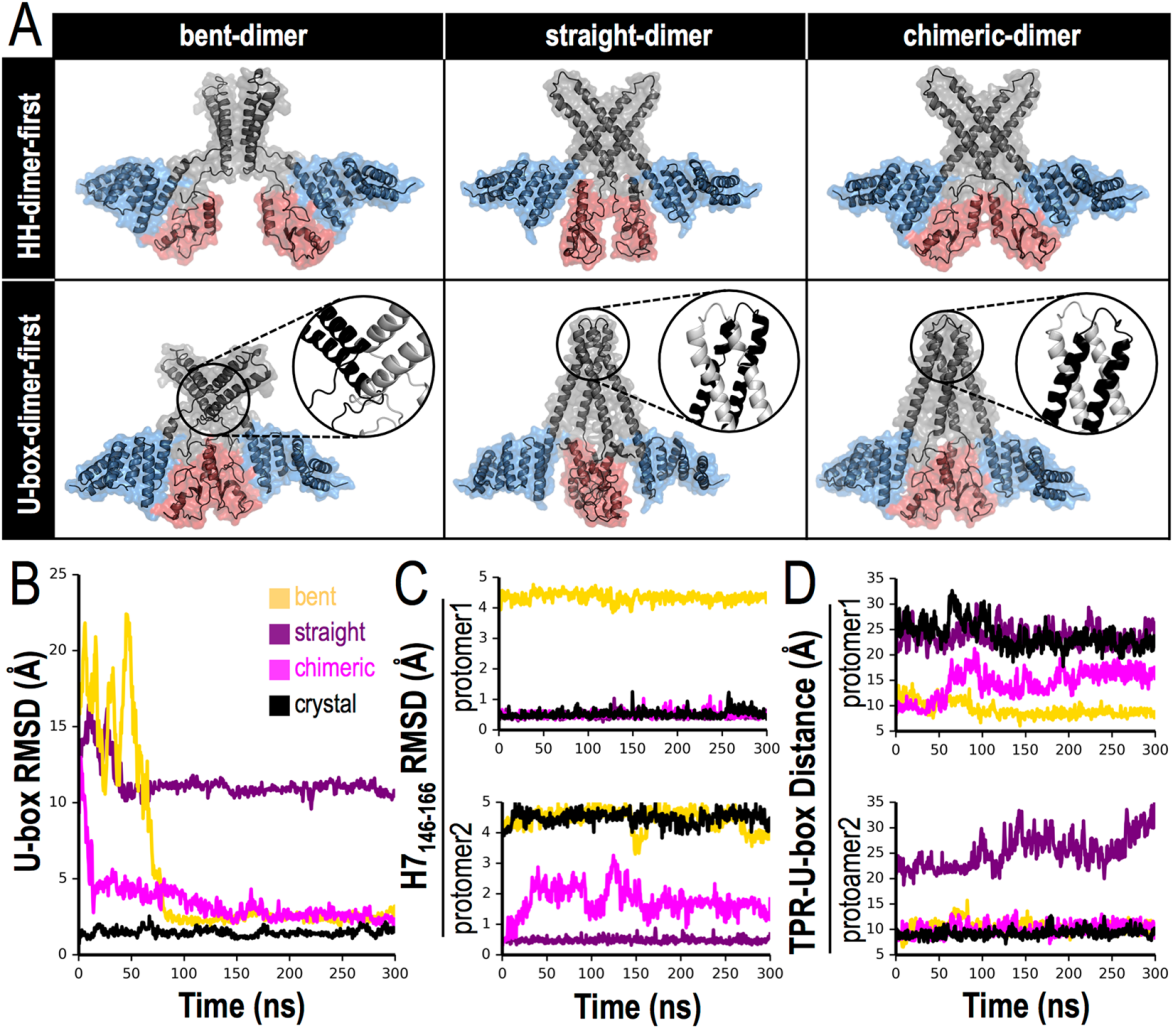
Assembly of CHIP monomers suggests that HH domains dimerize first. (**A**) CHIP assembly models. Upper/Lower rows show models where the corresponding HH/U-box dimers have been aligned as in the CHIP crystal for the bent, straight and chimeric monomers. Highlights in the lower row show structural clashes for bent and straight monomers and a mismatch for the chimeric dimer where the hydrophobic regions of the HH domains are on the outside. All the models are constructed using PyMOL 1.7. Mesh colors are as in Fig. 1. Representative MDS of HH-dimer-first models: (**B**) backbone RMSD of the U-box dimer relative to the crystal structure shows that the U-boxes from straight-dimer model does not dimerize; (**C**) Backbone RMSD of residues 146–166 in H7 for each protomer relative to their helical structure in the straight monomer (Fig. 2B) shows that the bent-dimer model does not form an extended H7; (**D**) TPR/U-box separation between Cα of residues TPR_R141_ and U-box_E239_, which interact in the bent monomer complex (Fig. 2A), shows that the straight-dimer never forms this complex and the bent-dimer never breaks them, whereas the chimeric-dimer breaks one complex and keeps the second intact, resembling the asymmetric structure of CHIP.

As shown in Fig. 3A (top), the HH-dimer-first models allow dimerization without steric clash. On the other hand, the HH domains clash in all three U-box-dimer-first models (Fig. 3A, bottom). Although the chimeric model shows the lowest degree of steric clash, the HH domains assemble incorrectly and expose their true dimerization interfaces to solvent. Based on this analysis and the expectation that the large hydrophobic patches of the HH domains provide a natural path for dimerization, we conclude that CHIP dimerization begins with HH dimer nucleation. It is interesting to note that a symmetric dimer formed by the two bent or chimeric monomers would be inactive, whereas a dimer formed by straight monomers may, in principle, bind two E2s.

### Can symmetric nucleation lead to an asymmetric dimer?

We next performed full atom unconstrained MDS in explicit solvent starting from each of three HH-dimer-first models shown in Fig. 3A. A representative snapshot of the resulting structures is shown in Supplementary Fig. S2. For the HH-bent-dimer model, the simulations indicate that the U-boxes dimerize on a time scale of >50 ns (Fig. 3B) and TPR-U-box domain packing remains stable in both protomers (Fig. 3D). Moreover, no extended H7 helix, as observed in the straight protomer of the CHIP crystal structure, forms in either protomer (Fig. 3C). The structure remains rather symmetric but flexible between the HH dimer and the TPR-bound U-box dimer. In the case of the HH-straight-dimer, the U-boxes do not dimerize during the simulation (Fig. 3B). Correspondingly, the TPRs remain co-axial with the extended helices (Fig. 3C) and do not interact with the U-boxes (Fig. 3D). This dimer is also relatively symmetric with flexible U-boxes that are separated from the TPRs by the C-terminus of each protomer. To test whether C-terminus insertion significantly slows down further structural rearrangements, we truncated the six C-terminal residues but found that the U-boxes still failed to dimerize. Within the time scale of our MDS, the straight helices allow little freedom for the U-boxes to properly accommodate a U-box dimer interface. Instead, a partial rotation moves the E2 binding sites into close proximity.

Finally, we simulated an HH-chimeric-dimer model in which the U-boxes are separated by more than 10 Å in the starting conformation. In these simulations, the U-boxes dimerize properly (Fig. 3B). Moreover, the extended H7 breaks in protomer 2 (see Fig. 3C), and the TPR-U-box complex is forcibly separated due to insertion of the C-terminal residues in protomer 1 (Fig. 3D). In principle, all pathways are allowed during CHIP dimerization, yet our simulations strongly suggest that not only the chimeric monomer and HH-chimeric-dimer model are more stable but also they are the kinetically favored path between a stable monomer and the asymmetric dimer. In contrast, in all other models tested (Supplementary Fig. S2) folding stalled in a symmetric structure. It should be noted that our longest MDS are about 600 ns, but that millisecond- or longer simulations may be required for these less likely initial configurations to progress towards the asymmetric dimer structure.

### Fast dissociation kinetics of TPR-U-box complex by the C-terminus is driven by U-box dimerization

**Figure 4:**
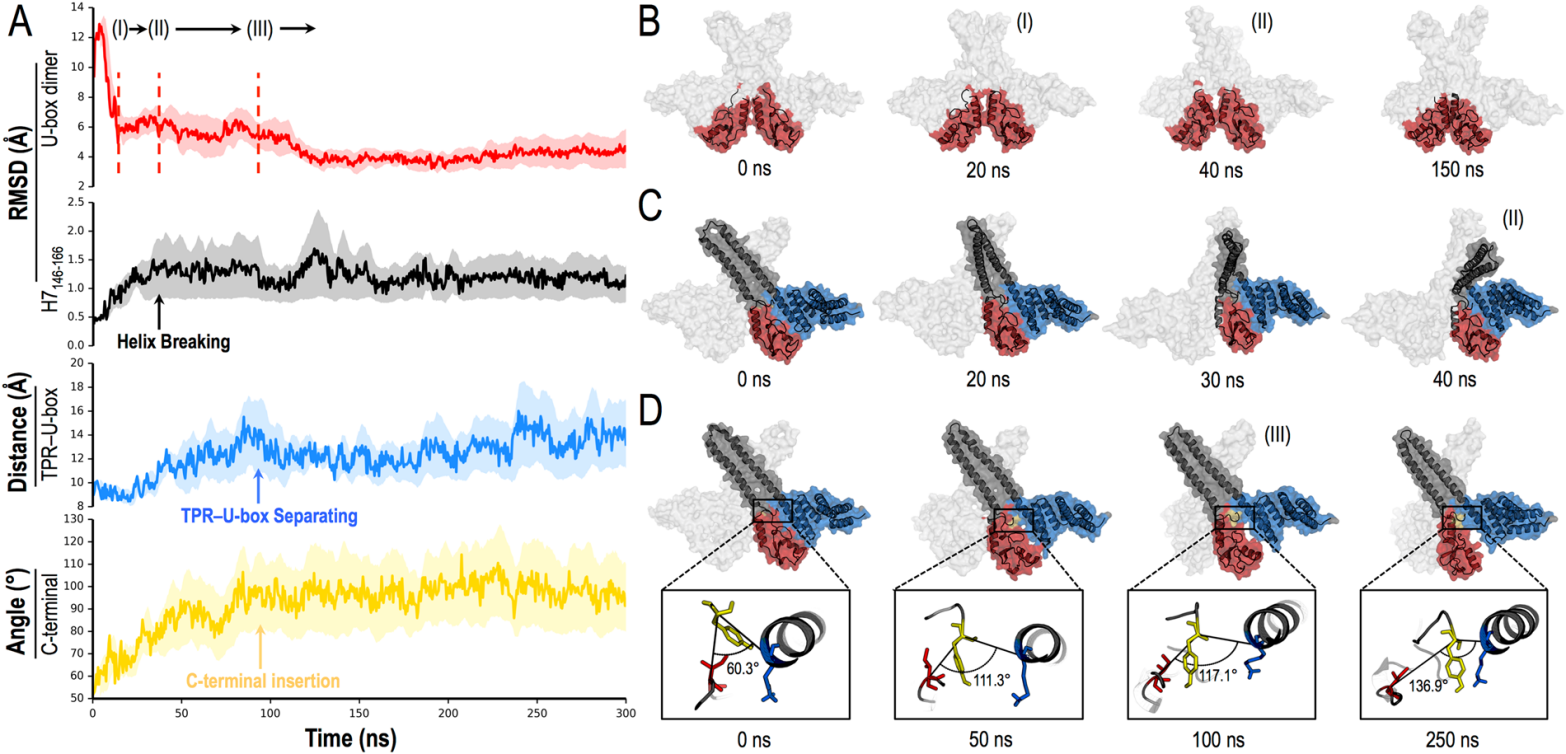
Molecular dynamics of HH-chimeric-dimer model reveals that U-box dimerization drives CHIP symmetry breaking. (**A**) Time progression of dimerization of HH-dimer-first chimeric model (see Fig. 3A) averaged over three independent runs (shades indicate standard deviation). Following the notation in Fig. 3B,C and D, we show: RMSD relative to the crystal structure of the U-box dimer, RMSD of the breaking region in H7, TPR-U-box separation, and the C-terminal angle between the vector joining the C-alpha’s of TPR_K145_ (blue stick) with C-terminus Y304 (yellow stick), and U-box_I228_ (red stick) and Y304, as a function of time. MDS snapshots at relevant time scales of (**B**) U-box dimerization, (**C**) breaking of H7, and (**D**) TPR–U-box separation with a magnified C-terminal insertion. Light gray mesh denotes the second monomer. See also Supplementary Video S1 of unconstrained MDS showing transition from symmetric to asymmetric dimer.

Starting from the symmetric HH-chimeric-dimer model, the transformation to the asymmetric structure follows a four-stage kinetic process driven by U-box dimerization. Namely, (a) rapid partial symmetric U-box dimerization is followed by (b) breaking of the extended helix in one protomer (i.e., breaking dimer symmetry), which leads to (c) the insertion of C-terminal residues into the U-box-TPR binding interface in the other protomer, and then (d) the partial U-box dimer assumes a native-like conformation. The resulting asymmetric dimer is in good agreement with the full CHIP crystal structure, yielding an overall backbone RMSD of 4.9 Å. The dynamics of this process is presented in Fig. 4.

Our MDS show that HH-domain dimerization in the chimeric model positions the U-boxes within interaction range, triggering their dimerization. In a fast time scale of ~20 ns, the U-boxes change from an RMSD of ~12 Å to ~6.0 Å relative to the U-box dimer in the CHIP crystal structure. However, since the U-boxes are bound to their corresponding TPRs, the dimers at both end of the long helices prevent the unwinding of the helices. Instead, dimerization brings together five consecutive basic amino acids (_221_KRKKR_227_) in the linker between the HH and U-box domains from each protomer. These basic sequences initially help to drive dimerization by interacting with U-box residues E_297_ and N_298_. However, the repulsive interactions of the positively charged linker residues eventually start leaning against _153_RRIH_158_ in H7, bending the extended helices. Ultimately, the electrostatic repulsion is relieved by breaking H7 of one protomer at this site in <50 ns (Fig. 4A,C). Partly due to interactions between the opposite linker and C-terminal negative charges (_301_EDY), the snapping of the helix forces the insertion of the C-terminus between the U-box and the TPR domain in the opposite protomer. This process takes about 40 ns, i.e., between 50 and 90 ns from the beginning of the simulation (Fig. 4A,D). Interestingly, it is not until all the stress created by the opposing interactions and helix breaking has relaxed and the asymmetry is established that U-box dimerization proceeds towards a high affinity complex between 90 and 120 ns (Fig. 4A,B).

### Deletion of the TPR domain leads to a symmetric CHIP structure

**Figure 5:**
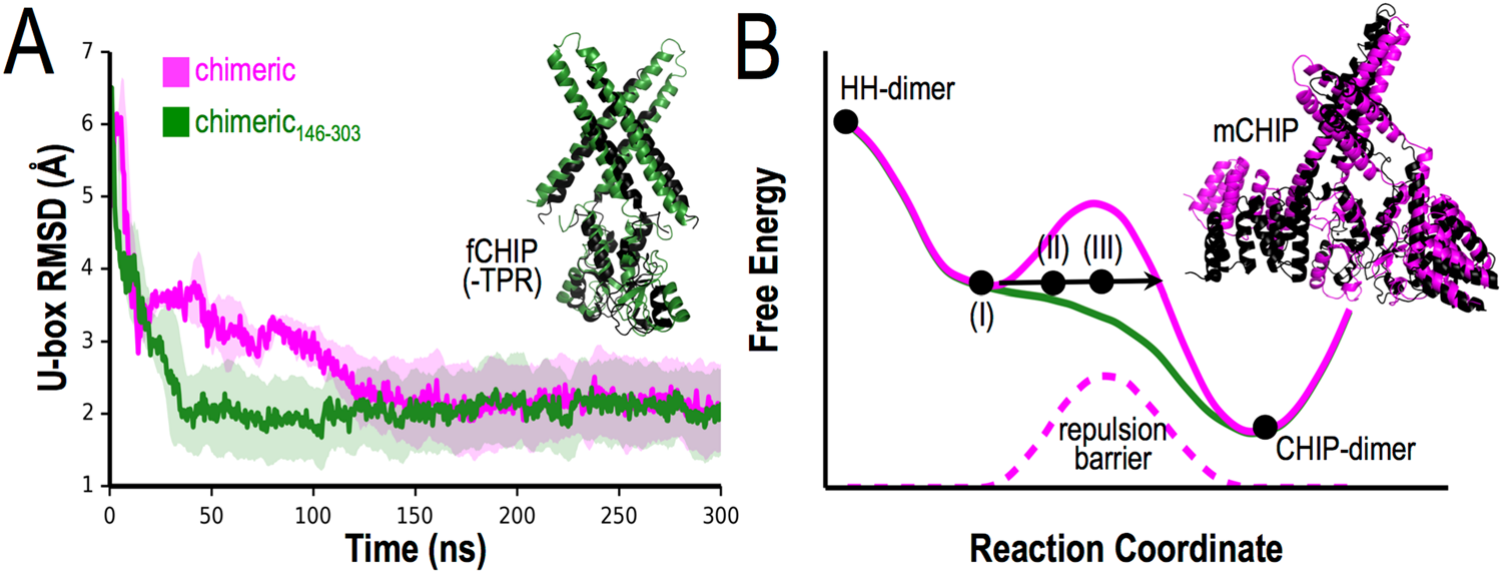
CHIP folds into a symmetric dimer in the absence of TPR. (**A**) Truncated HH-chimeric-dimer (CHIP_146–303_) folds into a symmetric conformation similar to fCHIP in the absence of TPR domains (overlap backbone RMSD of 3.9 Å relative to PDB 2F42 shown in black). RMSD of interacting helices in the U-box dimer as a function of time for CHIP (magenta) and truncated CHIP (green) averaged over three independent runs. (**B**) Sketch of the free energy landscape of U-box dimerization in the presence (magenta) and absence (green) of TPR. CHIP dimerization triggers a repulsive barrier that prevents the U-boxes to properly align until after H7 breaks, and the TPR and U-box detach (dashed line). The snapping of H7 generates an out-of-equilibrium pathway that overcomes the thermodynamic barrier imposed by the TPR-U-box interaction. Approximate loci of specific structural transitions observed in the MDS dimerization trajectories in Fig. 4B,C and D are indicated: (I) dimer recognition, (II) breaking of helix 7, and (III) C-terminal insertion.

The crystal structure of a Zebrafish CHIP construct (fCHIP) that lacks TPR domains (Fig. 1), is symmetric^12^, whereas full-length CHIP is asymmetric^8^. To determine whether our model predicts the formation of a symmetric dimer if the TPR domains are removed, we re-ran the HH-chimeric-dimer model with CHIP lacking residues 1–145. As expected, CHIP_146–303_ dimerized into a symmetric structure closely resembling the fCHIP crystal structure (Fig. 5A). The formation of this structure was guided by the same linker interactions described above. However, in the absence of the TPR domain the extended helices are free. Thus, the repulsive charges in the linker are not forced together and the U-boxes dimerize in ~50 ns (Fig. 5A).

### Removal of the C-terminus disrupts CHIP folding

**Figure 6:**
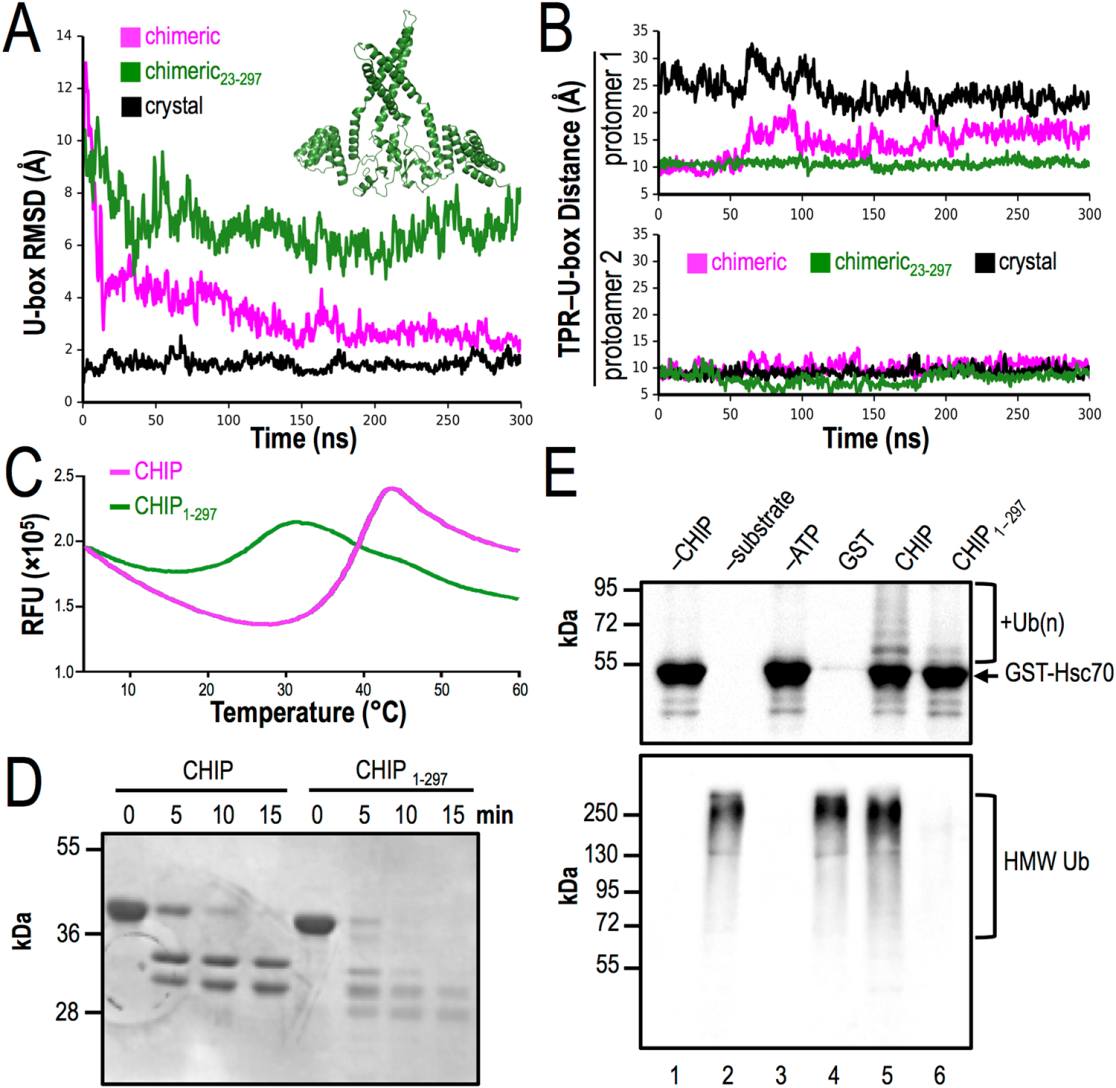
The C-terminus is critical for CHIP assembly and function. (**A**) Average of the three independent MDS show that U-boxes in truncated C-terminal CHIP_23–297_ can reach the recognition site, but do not progress to the full dimer within the time scale of our simulations. Inset shows a representative snapshot of CHIP_23–297_. (**B**) TPR-U-box separation shows that their binding interface is not disrupted, and both E2 sites remained buried. (**C**) Thermal unfolding assays that measure SYPRO dye binding (thermofluor) shows that the truncated mutant is destabilized by ~12.5 °C compared to full-length CHIP. (**D**) Limited proteolysis assay shows decreased stability of CHIP_1–297_. (**E**) CHIP_1–297_ substrate ubiquitination (top) and auto-ubiquitination (bottom) are significantly decreased.

We next performed MDS using a truncated form of CHIP, CHIP_23–297_, in which the six C-terminal residues were deleted. In these simulations, the dimerization process stalls after the U-boxes recapitulate fast recognition of the dimer interfaces observed in full-length CHIP (Fig. 6A). The truncated C-terminus no longer acts as a wedge, and the U-box-TPR interface is not disrupted within the time scale of our simulations (Fig. 6B). Instead, H7 is broken and attains a state that affords little room for the U-box recognition state to proceed toward the high affinity U-box dimer (inset Fig. 6A). Interestingly, this state tends to “lock in” a symmetric structure, which resembles the HH-bent-dimer and HH-straight-dimer models (see Supplementary Fig. S2).

Based on our simulations, the C-terminus plays a critical role in CHIP folding. We therefore expressed and purified a human CHIP construct lacking the six C-terminal amino acids, CHIP_1–297_. When compared to the purified full-length protein in thermal unfolding assays that measure SYPRO dye binding (thermofluor), the truncation mutant was destabilized by ~12.5 °C compared to full-length CHIP (Fig. 6C). These data are consistent with other reports on the relative thermal stability of wild type CHIP^24^, as well as the weakened U-box dimer interaction observed in our MDS when the C-terminus is truncated (Fig. 6A). A defect in the overall conformation was further evidenced by the decreased stability of the mutant protein in a limited proteolysis assay (Fig. 6D). To determine whether CHIP_1–297_ was substantially or fully unfolded, we compared circular dichroism (CD) spectra of CHIP_1–297_ to those of full-length CHIP. Consistent with the observed breaking of both H7 (see inset Fig. 6A), the CD spectra (Supplementary Fig. S3) show that CHIP_1–297_ contains substantial secondary structure content, but the calculated α-helical content is less than that of the full-length protein. In addition, the thermal transition in the overall structure of CHIP_1–297_ is not reflected in the temperature dependence of secondary structure content (Supplementary Fig. S3E). In other words, the transition at ~25 °C likely represents a loosening of the tertiary structure and disruption of the relative arrangement of domains within the dimer.

As the truncated protein appears to be more labile than full-length CHIP, we examined whether CHIP_1–297_ is catalytically active. Full-length and truncated forms of CHIP were incubated in a reaction containing E1, E2, ATP, ubiquitin, and a model substrate, the C-terminal client-binding domain of Hsc70 (Hsc70_395–646_; see Supplementary Methods). As shown in Fig. 6E, substrate ubiquitination was significantly abrogated in a reaction containing CHIP_1–297_, and CHIP_1–297_ auto-ubiquitination activity was absent. Together, these data support a critical role for the C-terminal residues in CHIP folding, dimerization, and stabilization.

## Discussion

The folding/binding pathway leading to an asymmetric dimer between identical proteins must overcome competing interactions. During the formation of such dimers, an intermolecular interaction between, for example, residue A_prot1_ and residue B_prot2_ is not duplicated between A_prot2_ and B_prot1_. Instead, protomer 2 must make distinct favorable contacts different from those of protomer 1. One might expect that overall dimer stability would suffer as a consequence of this frustrated landscape. Here, we report how this conundrum is resolved and describe for the first time an origin of symmetry breaking during the formation of a stable homodimer.

We first analyzed the stability of individual CHIP monomers (Fig. 2). The straight monomer exhibits a stable H7 helix and a rather flexible U-box, while the bent monomer exhibits a broken H7 and a stable TPR-U-box interaction. Intriguingly, a chimeric structure that combined the former (stable) H7 and the latter (interacting) TPR-U-box domains was more stable than either of the other two configurations. Although the structure of an isolated CHIP monomer has not been determined, Nikolay *et al*.^25^ showed that a chimeric dimer between a full-length CHIP monomer and an HH domain lacked ubiquitination activity. This finding is consistent with our predicted chimeric structure, in which the E2 binding site of the U-box is buried by the TPR domain. These data suggest that a CHIP monomer is inactive.

Two inactive monomers dimerize and undergo a series of conformational changes into a half-of-sites active dimer (see Supplemetary Video S1). We show that the most likely path to initiate CHIP dimerization is via the hydrophobic HH dimer interface (Fig. 3). Further validating our predicted structure of the chimeric monomer, we established that the HH dimer positions the U-box dimerization interfaces within interaction range, optimizing the assembly process. Namely, MDS demonstrate that in a time scale of ~150 ns (Fig. 4) U-box dimerization triggers symmetry breaking of the dimer and disrupts one of the TPR-U-box complexes. The latter event exposes an E2 binding site, resulting in a ligase competent to ubiquitinate exogenous substrates.

The TPR-U-box interactions that also involve some contacts with H7 are predicted to be stronger than those within the individual dimer interfaces, ΔG_int_ = −20 kcal/mol^23^. This presumes that the TPR and U-box might have a dissociation time scale of milliseconds or longer. Strikingly, the novel symmetry breaking mechanism uncovered in this report would instead disrupt this interface in <100 ns (Fig. 4). Following the formation of an HH-chimeric-dimer, the U-boxes start to dimerize and in ~20 ns they reach a low free energy “recognition” state. This state is a prelude to the more subtle structural rearrangements that form the higher affinity U-box dimer present in CHIP crystal structures. U-box-U-box interaction drives the extended HH helices together, which push against the positively charged linker (_221_KRKKR_227_) situated between the TPR and HH domains. It is important to note that the helices are held at both ends by the HH and U-box dimers, which prevents the smooth unwinding of the helices. Instead, repulsive interactions between the linkers and _153_RRIH_158_ in H7 eventually snap one of the extended helices at this site. This motion wedges the C-terminus of the other protomer against the binding interface of the corresponding TPR and U-box domains, splitting the complex in ~100 ns. It is only after these transitions are completed and the structure is relieved of competing interactions that U-box dimerization process can proceed.

Our data suggest that the binding free energy of the partial U-box dimer provides a reservoir to resolve conflicting interactions in the linker regions and occlusion of the E2 binding sites by the TPR domains (Fig. 5B). Such interactions present a barrier in the U-box dimerization pathway. Instead of letting thermodynamics (i.e., large thermal fluctuations) control the activation of CHIP, we show for the first time that opposing interactions can generate a force that leads to the kinetic control of conformational activation (Fig. 5B). MDS further validate our findings by demonstrating that removing the TPR from the HH-chimeric-dimer eliminates this barrier and accommodates tighter packing of the U-boxes, which in turn leads to a thermodynamically stable symmetric dimer. The latter is in full agreement with the fCHIP crystal structure that lacks the TPR domains.

We also analyzed the critical role played by the wedge or lever action of the C-terminal segment, using both MDS and *in vitro* experiments. Specifically, we demonstrate that CHIP_1–297_–dependent substrate ubiquitination and auto-ubiquitination were significantly reduced relative to wild-type CHIP (Fig. 6). Simulations show that in the absence of the C-terminal segment, intraprotomer TPR-U-box packing is stable, rendering CHIP_1–297_ mostly inactive by hindering the E2 binding site. The initial stages of U-box dimerization still break the extended H7 at _153_RRIH_158_. However, consistent with reduced helical content shown in the CHIP_1–297_ CD spectra (Supplementary Fig. S3), our simulations indicate that H7 breaks in *both* monomers of CHIP_1–297_. Engaged with their TPR domains, the U-boxes lack the freedom to easily rearrange into a higher affinity dimer, stalling the dimerization process around the dimer recognition state (Fig. 5). The incomplete U-box dimer should accordingly weaken the stability of the CHIP_1–297_ dimer, which is consistent with a shift in the thermofluor measurements and increased susceptibility to exogenous protease (Fig. 6).

Activation by dimerization and phosphorylation are vital during signal transduction and can bypass the long time scales entailed by thermodynamic control of large scale structural reorganization and/or dissociation of protein domains^26^. Yet, the detailed molecular mechanisms of how ATP-hydrolysis achieves these events are unclear. To our knowledge, our study represents the first unbiased full atom MDS to show how a homodimer can assemble into an asymmetric structure and generate a force to kinetically disrupt a domain-domain binding interface and activate an enzyme. The symmetry breaking pathway, which involves long helices and dimerization events common to many regulatory proteins reveals key structural transitions leading to a half-of-sites active enzyme.

Finally, we propose that asymmetry is functionally connected to CHIP autoubiquitination when substrate proteins are absent^11, 27, 28^, as well as to other E3 ligases with similar domain architecture. The most prominent CHIP autoubiquitination site is K_2_^29, 30^, located within a flexible ~25-residue N-terminal segment of CHIP. Our modeling suggests that K_2_ of the straight (active) protomer is the only lysine that can readily access the active site of a bound E2 enzyme (not shown). Hence, the straight protomer may more likely be autoubiquitinated and targeted for degradation than the bent protomer. Another, non-mutually exclusive possibility is that asymmetry is a by-product of assembling a ubiquitin ligase. Namely, as a monomer, CHIP may block E2 binding, preventing premature CHIP degradation until a fully formed dimer is assembled. Experimental tests of these models are in progress.

## Methods

A detailed description of materials and methods in given in Supplementary Information. Briefly, simulations were carried out using standard full atom MDS in explicit solvent. MDS were carried out in triplicate. Analysis of independent runs are shown in Supplementary Figures S4, S5 and S6. MD initial structures were built from available crystal structures and are available upon request. Video of representative MDS of CHIP dimerization is shown in Supplementary Video S1. Methods used for *in vitro* experiments including CHIP thermostability and activity measurements are described in detail in the Supplementary Information.

## Electronic supplementary material

Supplementary Information

Asymmetric dimerization of CHIP
