# Supplementary material for: Symmetry breaking during homodimeric assembly activates an E3 ubiquitin ligase
Source: Sci Rep. 2017 May 11;7:1789. doi: 10.1038/s41598-017-01880-4 (PMC5431976; doi:10.1038/s41598-017-01880-4)
Supplement: Supplementary file 1 — Supplementary Information [file 41598_2017_1880_MOESM1_ESM.pdf]

Supplementary Information

Symmetry breaking during homodimeric assembly activates an E3 ubiquitin ligase

Zhaofeng Ye[1,4], Patrick G. Needham[2], Samuel K. Estabrooks[2], Susan K. Whitaker[3], Brandon L. Garcia[3], Saurav Misra[3], Jeffrey L. Brodsky[2], Carlos J. Camacho[1]*

[1]Department of Computational and Systems Biology and [2]Department of Biological Sciences, University of Pittsburgh, Pittsburgh, PA 15206. [3]Dept. of Biochemistry and Molecular Biophysics, Kansas State University, Manhattan, KS 66506. [4]School of Medicine, Tsinghua University, Beijing, China 100084.

*corresponding author: ccamacho@pitt.edu

Materials and Methods

Structure Preparation. All structural alignments were carried out using PyMOL 1,7 [1]. Straight monomer (Fig. 2B) and bent monomer (Fig. 2A) were extracted from PDB 2C2L (chain B and chain A). The chimeric monomer was constructed using 2C2L chain A residues 24-145, chain B 146-223 and chain A 224-304. HH-first-dimer models (HH-bent-dimer, HH-straight-dimer and HH-chimeric-dimer) were constructed by aligning HH residues 161-219 from two monomers to the equivalent residues in 2C2L chain A and chain B. Similarly, U-box-first-dimer models (U-box-bent-dimer, U-box-straight-dimer and U-box-chimeric-dimer) were constructed by aligning U-box residues 229-304 from two monomers to the equivalent residues in 2C2L chain A and chain B.

Simulation Protocol. The molecular dynamics simulations (MDS) were run with pmemd.cuda[2] from AMBER14[3] using AMBER ff12SB force field. We used the tLEaP module from AMBER14 to center each individual system (i.e., monomer, dimer and chimeras) on an octahedral TIP3P water box[4] with a 15 Å distance from the protein surface to the box edges and closeness parameter of 0.75 Å. The system was neutralized by adding the appropriate number of Na^+ ions. Thus, one MDS of, say, the CHIP monomer with a straight helix 7 entailed 48,251 water molecules and 9 Na^+ ions in a 1,596,532 Å^3 volume. In the first energy minimization step, the solute was fixed and we performed two rounds of solvent relaxation through 2,500 cycles of steepest descent followed by 2,500 cycles of conjugate gradient minimization. After minimization, we performed a 50 ps constant volume simulation to raise the temperature of the system to 300 K. Temperature scaling was achieved using the Langevin thermostat[5] with a collision frequency of 1.0 ps^{-1}. We then ran a 50 ps constant pressure simulation at 300 K using the same temperature scaling scheme. Pressure was maintained at 1 bar (0.987 atm) using periodic boundary conditions with isotropic position scaling and the Berendsen thermostat[6] with a pressure relaxation time of 2.0 ps. Both the temperature and pressure equilibration MDS used an integration timestep of 2 fs, and coordinates were written to file every 250 steps. Solute heavy atoms were fixed using a harmonic restraint weight of 500.0 kcal/molA^2, and bonds involving hydrogens were constrained at constant length using the SHAKE algorithm[7]. The non-bonded interaction cutoff was 8 Å. Initial velocities were generated randomly from a Maxwell distribution at 300 K. All production runs were performed without constraints for 200 ~ 300 ns, and extended for another 200 ~ 300 ns, as noted.

Purification of CHIP and CHIP$_{1\text{-}297}$. The full length human CHIP sequence with N-terminal polyhistidine and V5 affinity tags was PCR amplified from a pET151/D-TOPO-CHIP construct (Invitrogen) using the primers Nde1CHIP 5'GATATACATATGCATCATCACCATCAC3' and BamHICHIP 5'TCTATGGATCCTTAATTCTCAGAGATGAATGCGTC3' (IDT). The resulting PCR product generates a truncated CHIP coding sequence lacking the final six amino acids (CHIP$_{1\text{-}297}$). The DNA corresponding to the truncated CHIP mutant was digested with *NdeI* and *BamHI* (Fermentas), gel purified, and ligated into pET21a (Novagen). Clones were isolated and sequenced to confirm the correct truncation and the resulting plasmid was introduced into BL21(DE3) *E. coli* (New England Biolabs) by standard transformation techniques.

Full-length CHIP and CHIP$_{1\text{-}297}$ were purified by the following procedure. BL21(DE3) cells carrying the CHIP expression vector were grown from a single colony in LB medium

supplemented with 50 μg/ml ampicillin at 30ºC until an A_{260} of 0.6 was reached. IPTG was added to the growth media at a final concentration of 1.0 mM and growth was continued for 2 h at 30ºC. Induced cells were collected by centrifugation and cell pellets were stored at -80ºC.

To purify CHIP, cell pellets were thawed on ice and suspended in lysis buffer (50 mM $NaPO_4$, pH 8.0, 300 mM NaCl, 10 mM imidazole, 3 mM 2-mercaptoethanol, 0.25% TritonX-100, 1 mM PMSF, 2 mg/ml leupeptin, 2 mg/ml pepstatin A). A freshly made aliquot of chicken egg lysozyme (Sigma) was added to a final concentration of 1 mg/ml and the suspension was incubated on ice for 30 min to begin cell lysis. The cell suspension was then subjected to sonication for three one minute bursts with cooling on ice. The cell lysate was clarified by centrifugation for 10 min at 12,000 RPM in a Sorvall SS34 rotor at 4ºC. The lysate was loaded on a 5ml Nickel NTA column (Qiagen) and allowed to drip through by gravity flow. The column was washed with an additional 30 ml of lysis buffer (10 mM imidazole) and then 30 ml of lysis buffer containing 30 mM imidazole. CHIP was eluted from the column in lysis buffer containing 200 mM imidazole. CHIP-containing fractions were identified and pooled after SDS-PAGE and staining with Coomassie Brilliant Blue. Purified CHIP was subjected to overnight dialysis in 50 mM HEPES, pH 7.5, 150 mM NaCl, 20% glycerol, and aliquots were snap frozen in liquid nitrogen and stored at -80ºC.

Biochemical Assays

Thermal denaturation Purified CHIP protein was diluted in 50 mM HEPES, pH 7.5, 150 mM NaCl to a final concentration of 5 μM. SYPRO Orange (5000X, Life Technologies) was added at to a 20X concentration. Samples (20 μl) were assembled in triplicate in 96 well plates at 4ºC and loaded immediately into a StepOnePlus real time PCR machine (Applied Biosystems) and relative fluorescence was measured at 575 nm from 4ºC to 60ºC in 0.3ºC increments with a 15 sec hold at each temperature. The data obtained for CHIP and $CHIP_{1\text{-}297}$ were normalized to one another to highlight the difference in thermal stability.

Limited Proteolysis Purified CHIP protein (15 g) in a 25 μl reaction with 100 mM Tris, pH 8.0 was incubated at 26ºC. 10 ng Bovine trypsin (Sigma) prepared in 1 mM HCl was added to each reaction and 6 μl aliquots were removed at 0, 5, 10, and 15 min time points into an equal volume of 2X SDS-PAGE sample buffer supplemented with 5 mM PMSF, 2% 2-mercaptoethanol. Tubes were placed immediately in a 98ºC heat block for 10 min. Reactions were cooled and evaluated by SDS-PAGE.

Circular Dichroism spectrometry Purified CHIP and $CHIP_{1\text{-}297}$ were dialyzed into 50 mM sodium phosphate, pH 7.8 overnight and diluted to final concentrations of 5.2 μM (CHIP) and 3.7 μM ($CHIP_{1\text{-}297}$), respectively. CD spectra were collected from 100 μL samples in a 0.2 mm pathlength jacketed cuvette (Starna). Spectra were collected on a Jasco J-815 CD spectrometer equipped with circulating bath, from samples at 10˚C to 80˚C at 5˚C intervals. Five scans from 185-260 nm were averaged for each spectrum. Spectra were corrected in SpectraManager software by subtracting the spectrum of 100 μL of buffer collected at the corresponding temperature. The corrected spectra were further smoothened using a Savitzky-Golay filter and the smoothened spectra were analyzed for secondary structure composition using BestSel [8]. CHIP spectra at low

temperature corresponded well in terms of secondary structure composition to estimated values calculated from the crystal structure of mCHIP (PDB 2C2L [9]).

***In Vitro* Ubiquitination Assays** To compare the relative activities of CHIP and $CHIP_{1-297}$, the purified CHIP proteins were tested for autoubiquitination and ubiquitination of a protein substrate *in vitro*. Ubiquitination reaction mixes were prepared first by combining 0.125 μM E1 (Ube1) and 1 μM E2 (UbcH5b) (Boston Biochem), 200 μM ubiquitin (Sigma), and the appropriate volume of 10X reaction buffer (50 mM HEPES, pH 7.0, 50 mM NaCl, 20 mM ATP, 40 mM $MgCl_2$), followed by a 30 min incubation at 37°C. In parallel, a total of 3 μM of purified CHIP was combined on ice with 10 μM of the purified ubiquitination substrate, a GST fusion of the Hsc70 substrate recognition domain (GST-$Hsc70_{395-646}$) [10], in 50 mM HEPES, pH 7.0, 50 mM NaCl. Control reactions were also set up in the absence of either CHIP, the substrate, or ATP, or to test the substitution of GST in place of the substrate. After addition of the two mixtures, reactions were incubated for 1 hr at 20°C, and then stopped by the addition of SDS-PAGE sample buffer supplemented with 50 mM EDTA. The quenched reactions were resolved by SDS-PAGE, transferred onto nitrocellulose membranes, and probed with either anti-GST HRP-conjugated antibody (Abcam) or anti-ubiquitin (Santa Cruz), which was detected with horse anti-mouse-HRP antibody (Cell Signaling). Concentrations provided for all purified proteins used in the reactions reflect their final reaction concentrations.

Supplementary Figure and Video Captions

Figure S1. Folding free energy (G) estimations of straight, bent and chimeric model suggest that the latter is more stable. Calculations were made using amber mm_pbsa in the AMBER package with generalized Born (GB) force field. Although values are highly dependent on dielectric constant and length of simulation, the relative stability of the chimeric monomer is consistent with the stable long helix 7 and tight TPR-U-box interaction observed in our MDS.

Figure S2. Representative snapshots of the final conformations for the assembly models. (A), (B) and **(C)** are for the HH-dimer-first models. **(D)** and **(E)** for the C-terminal truncated HH-straight-dimer model and HH-chimeric-dimer model, respectively. Blue mesh depicts the TPR domains. Grey mesh indicates the HH dimers. Red mesh shows U-box dimers.

Figure S3. Structural stability of CHIP and CHIP$_{1\text{-}297}$. (A)(B) CD spectra and **(C)(D)** secondary structure content of CHIP and CHIP$_{1\text{-}297}$ at 10˚C (red) to 80˚C (deep purle) in 5˚C intervals. Although CHIP$_{1\text{-}297}$ has lower overall helical content than CHIP, secondary structure fractions and **(E)** CD signal at 222 nm show that a secondary structures of both species change only with a broad transition centered above 40˚C.

Figure S4. Duplicates of MDS for monomer stability in addition to Fig. 2DE. Of note, the TPR and U-box in (B) do not make contact in a conformation similar to the bent monomer, and further structural rearrangements are slow relative to the scale of our simulations. Both in (A) and (B), the bent helix remains flexible and bent as in the crystal monomer (Fig. 2A).[11]

Figure S5. Duplicates of MDS for HH-dimer-first models in addition to Fig. 3BCD. During the MDS of the HH-bent-dimer in Set 3, the U-box-TPR domains from each monomer did not run into each other but dangled away (See, also, HH-bent-dimer in Fig. 3A).

Figure S6. Duplicates of MDS for truncated HH-chimeric$_{23\text{-}297}$-dimer in addition to Fig. 6AB. During the MDS of the truncated HH-chimeric$_{23\text{-}297}$-dimer in Set 3, the U-boxes sampled a close to native interface, the TPR-U-box did not split as much as in the full HH-chimeric-dimer MDS, and the E2 binding site remained occluded.

Video S1. Representative unconstrained MDS of CHIP dimerization. Initial structure is the symmetric HH dimer of the chimeric model. Of note are the following transitions: rapid U-box dimerization of the U-box, followed by (right) helix breaking, and halfway through the MDS a gap opens between the HH and U-box domains in the left side. Movie is 250 ns.

1 Schrödinger, L. The PyMOL Molecular Graphics System, Version 1.7. 4 Schrödinger, LLC.
2 Gotz, A. W. *et al*. Routine Microsecond Molecular Dynamics Simulations with AMBER on GPUs. 1. Generalized Born. *J. Chem. Theory Comput.* **8**, 1542-1555, doi:10.1021/ct200909j (2012).
3 Case, D. *et al*. Amber 14. (2014).
4 Jorgensen, W. L., Chandrasekhar, J., Madura, J. D., Impey, R. W. & Klein, M. L. Comparison of Simple Potential Functions for Simulating Liquid Water. *J. Chem. Phys.* **79**, 926-935, doi:Doi 10.1063/1.445869 (1983).
5 Loncharich, R. J., Brooks, B. R. & Pastor, R. W. Langevin Dynamics of Peptides - the Frictional Dependence of Isomerization Rates of N-Acetylalanyl-N'-Methylamide. *Biopolymers* **32**, 523-535, doi:DOI 10.1002/bip.360320508 (1992).
6 Berendsen, H. J. C., Postma, J. P. M., Vangunsteren, W. F., Dinola, A. & Haak, J. R. Molecular-Dynamics with Coupling to an External Bath. *J. Chem. Phys.* **81**, 3684-3690, doi:Doi 10.1063/1.448118 (1984).
7 Ryckaert, J. P., Ciccotti, G. & Berendsen, H. J. C. Numerical-Integration of Cartesian Equations of Motion of a System with Constraints - Molecular-Dynamics of N-Alkanes. *J Comput Phys* **23**, 327-341, doi:Doi 10.1016/0021-9991(77)90098-5 (1977).
8 Micsonai, A. *et al*. Accurate secondary structure prediction and fold recognition for circular dichroism spectroscopy. *Proc Natl Acad Sci U S A* **112**, E3095-3103, doi:10.1073/pnas.1500851112 (2015).
9 Zhang, M. *et al*. Chaperoned ubiquitylation--crystal structures of the CHIP U box E3 ubiquitin ligase and a CHIP-Ubc13-Uev1a complex. *Mol. Cell* **20**, 525-538, doi:10.1016/j.molcel.2005.09.023 (2005).
10 Zhang, H. *et al*. A bipartite interaction between Hsp70 and CHIP regulates ubiquitination of chaperoned client proteins. *Structure* **23**, 472-482, doi:10.1016/j.str.2015.01.003 (2015).
11 Clarke, D. T., Doig, A. J., Stapley, B. J. & Jones, G. R. The alpha-helix folds on the millisecond time scale. *Proc. Natl. Acad. Sci. U. S. A.* **96**, 7232-7237 (1999).

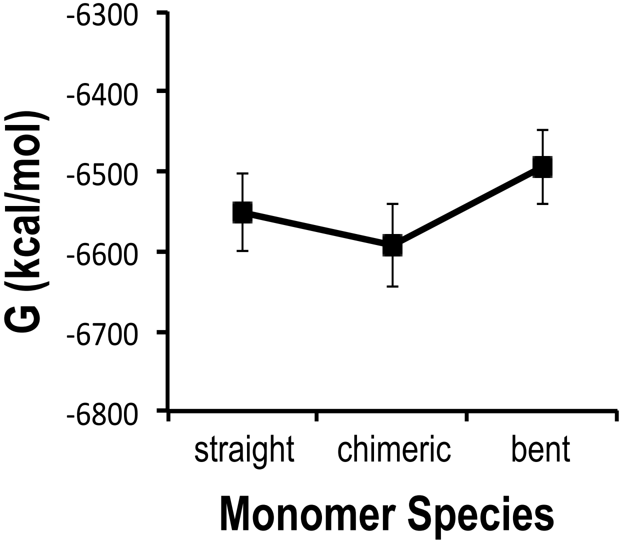
-6300
-6400
-6500
-6600
-6700
-6800
G (kcal/mol)
straight
chimeric
bent
Monomer Species

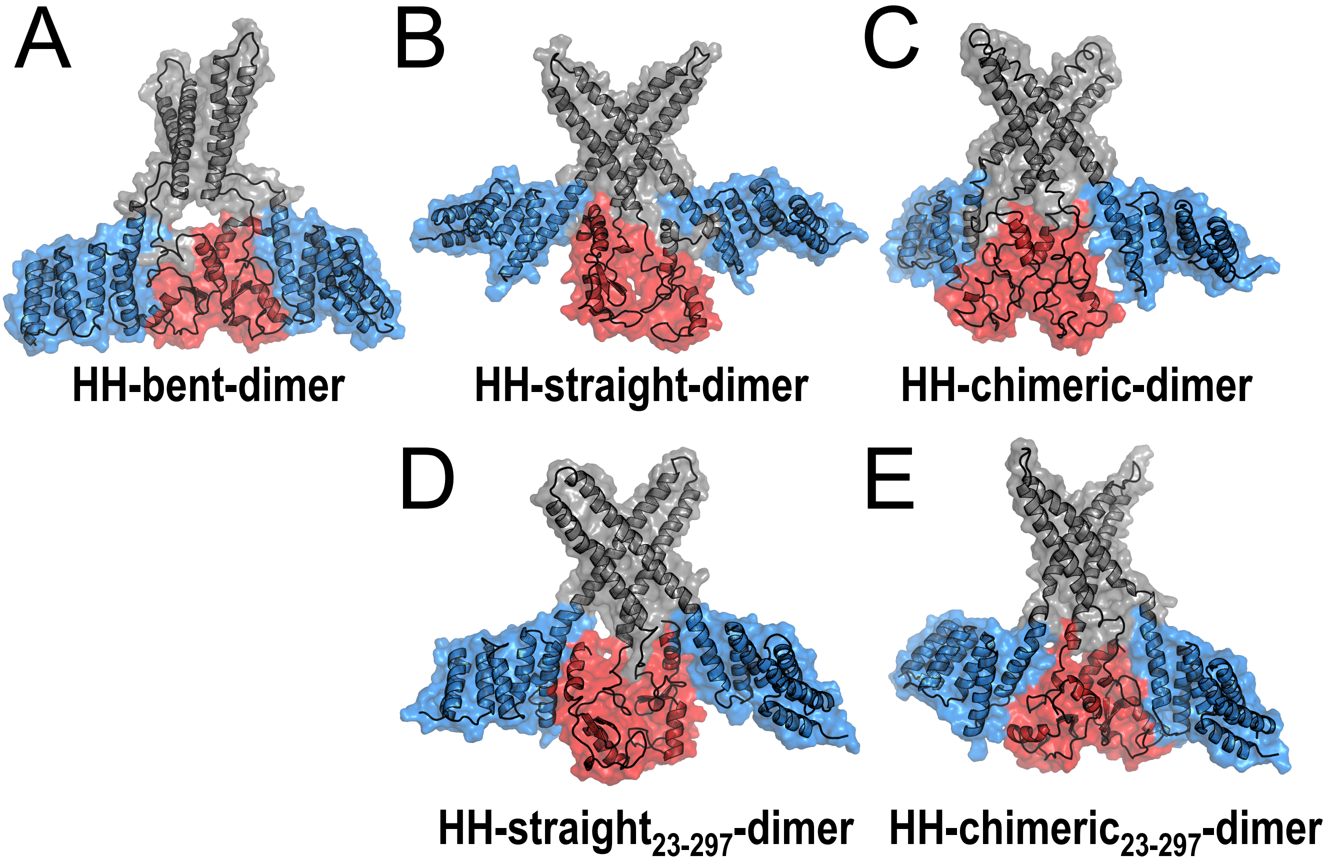
A
B
C
HH-bent-dimer
HH-straight-dimer
HH-chimeric-dimer
D
E
HH-straight$_{23-297}$-dimer
HH-chimeric$_{23-297}$-dimer

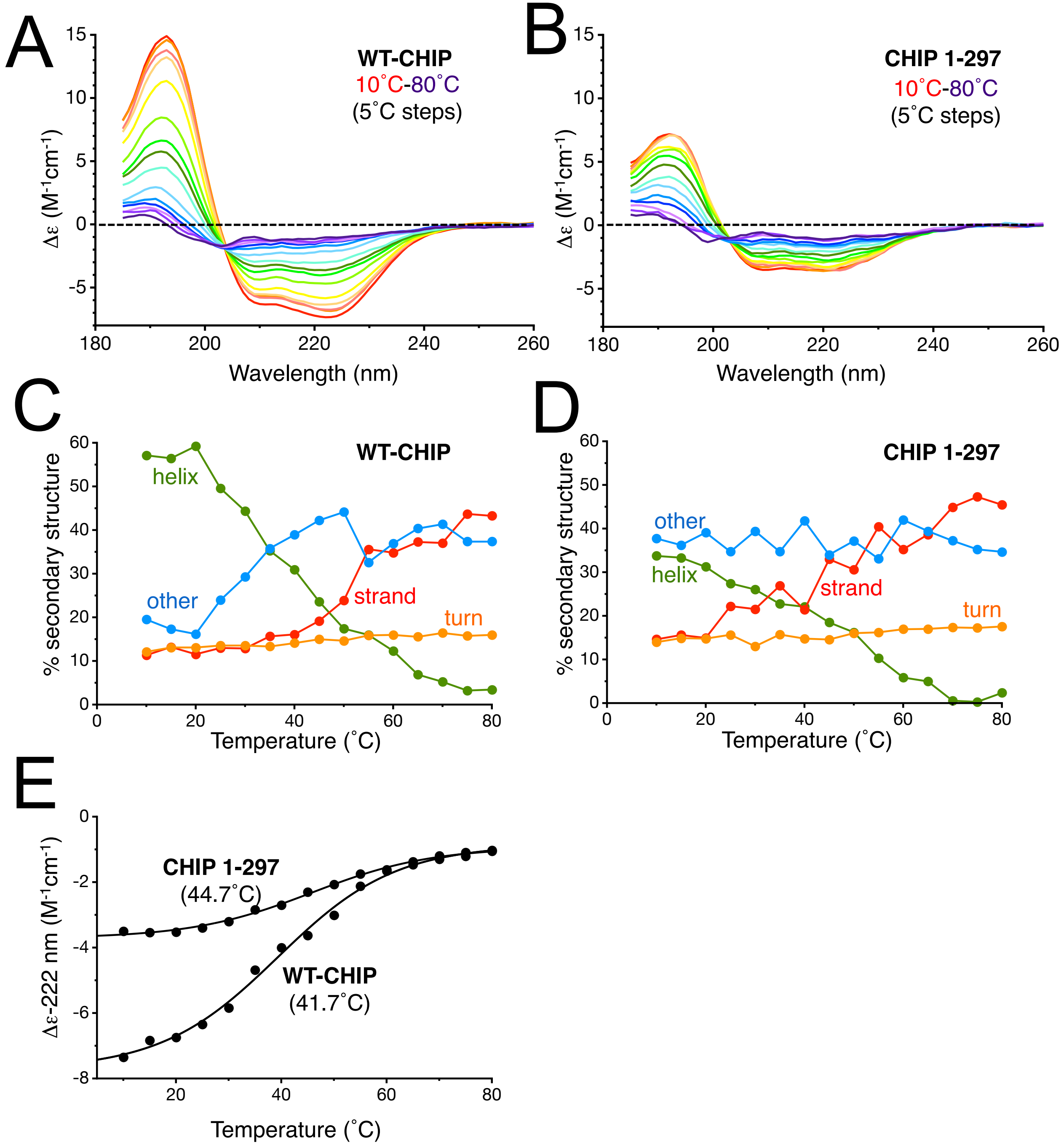
A
WT-CHIP
10˚C-80˚C
(5˚C steps)
Δε (M-1cm-1)
Wavelength (nm)
B
CHIP 1-297
10˚C-80˚C
(5˚C steps)
C
WT-CHIP
helix
other
strand
turn
% secondary structure
Temperature (˚C)
D
CHIP 1-297
other
helix
strand
turn
E
CHIP 1-297
(44.7˚C)
WT-CHIP
(41.7˚C)
Δε-222 nm (M-1cm-1)
Temperature (˚C)

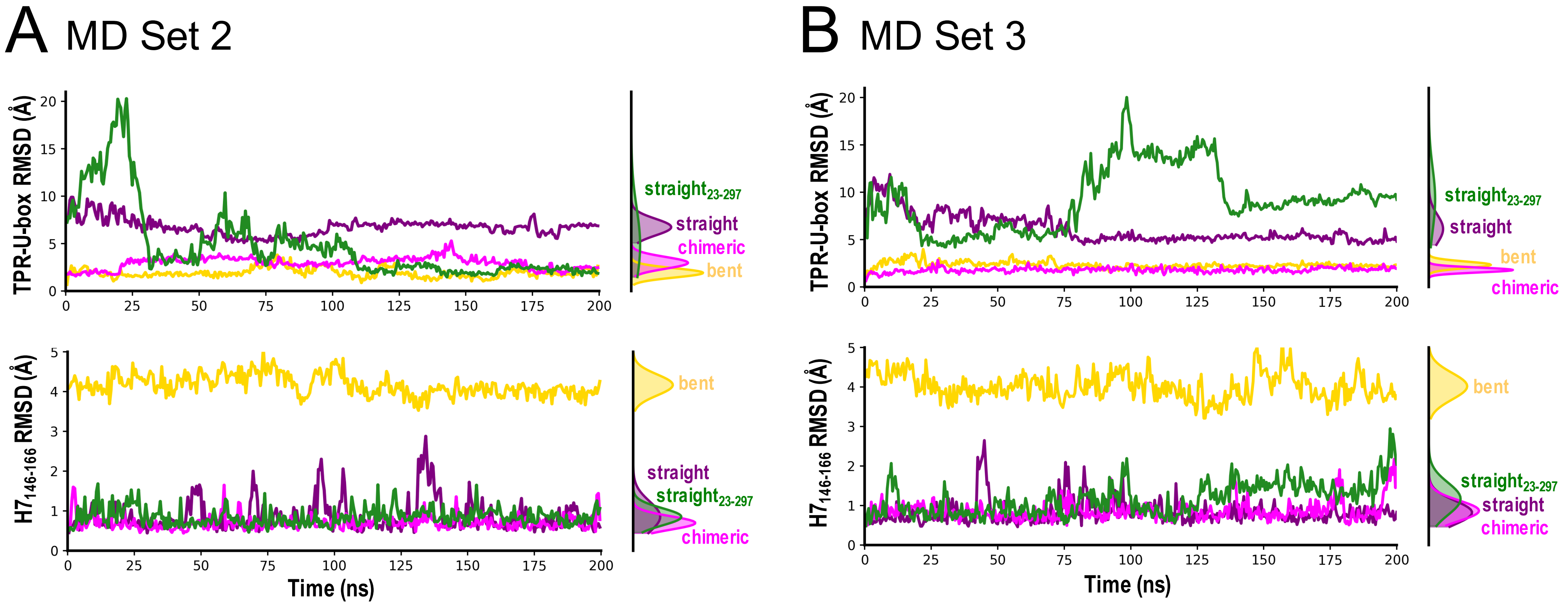

A MD Set 2
B MD Set 3
TPR-U-box RMSD (Å)
H7146-166 RMSD (Å)
Time (ns)
straight23-297
straight
chimeric
bent

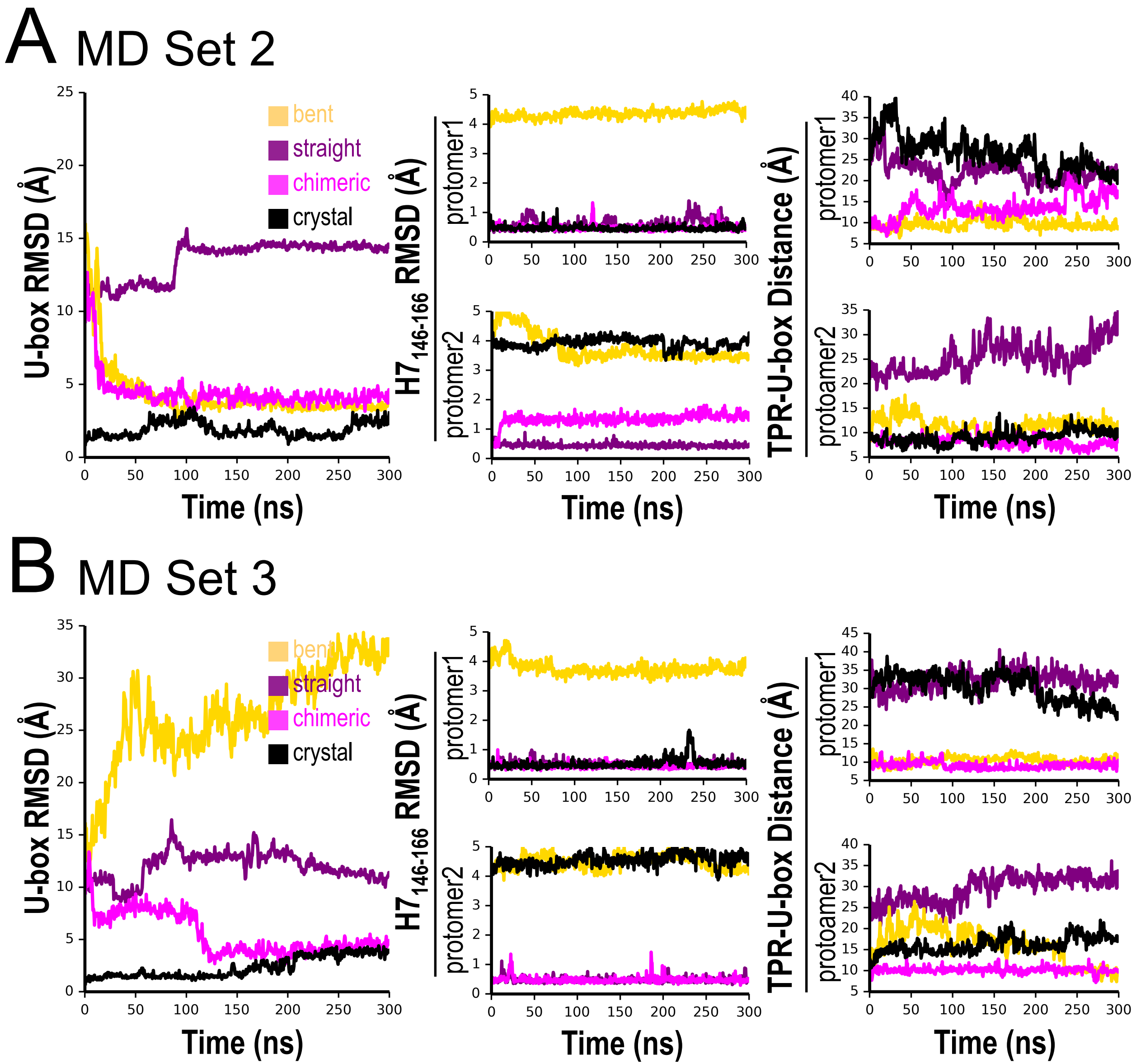
A MD Set 2
B MD Set 3
U-box RMSD (Å)
bent
straight
chimeric
crystal
$H7_{146\text{-}166}$ RMSD (Å)
protomer1
protomer2
TPR-U-box Distance (Å)
protoamer2
Time (ns)

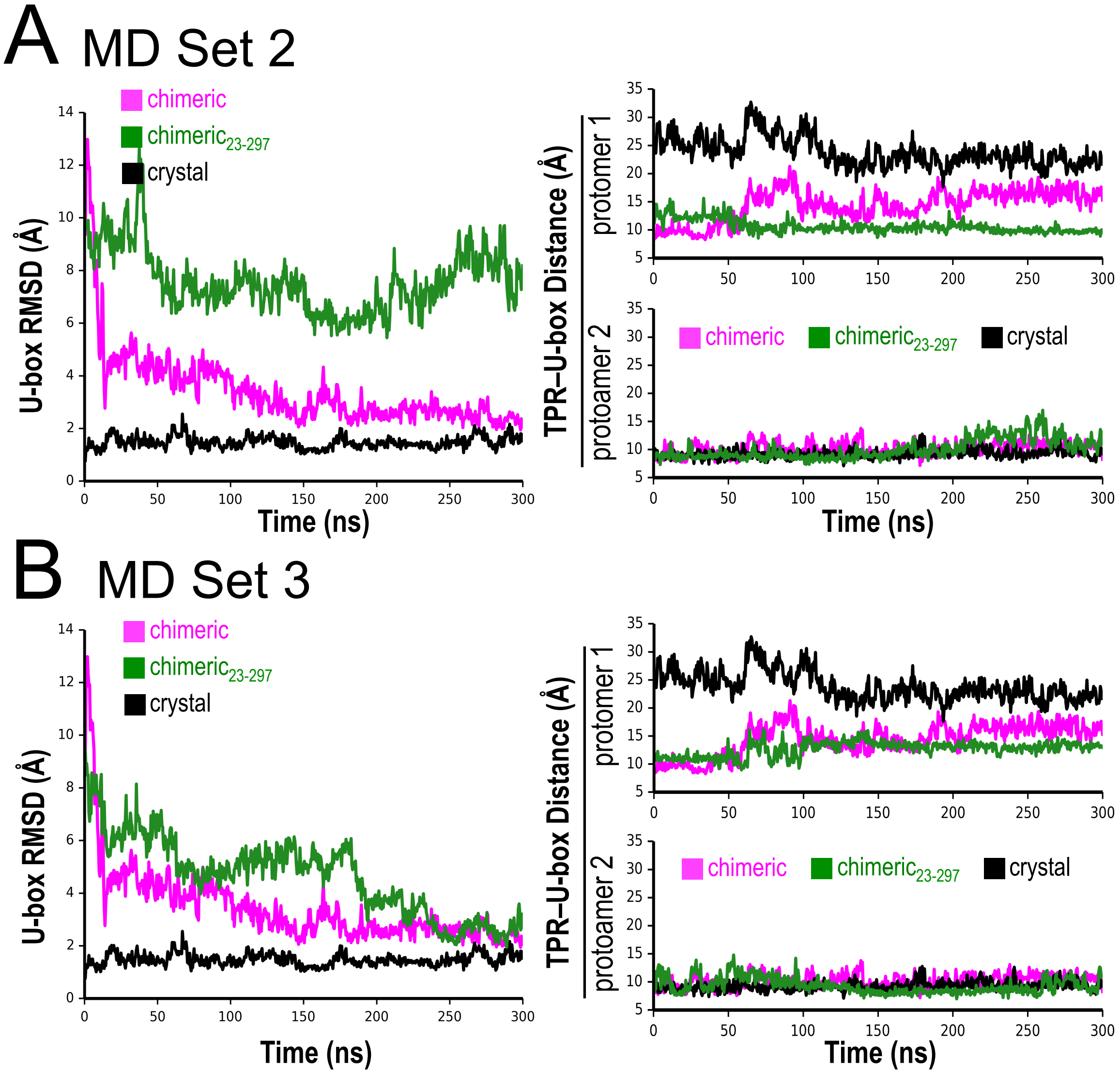
A
MD Set 2
chimeric
chimeric23-297
crystal
U-box RMSD (Å)
Time (ns)
TPR–U-box Distance (Å)
protomer 1
protoamer 2
B
MD Set 3
